# A Rare Presentation of Disorder of Sex Development

**DOI:** 10.7759/cureus.12782

**Published:** 2021-01-19

**Authors:** Sara Ashfaq, Ahmed Siddiqui, Waqas Shafiq, Umal Azmat

**Affiliations:** 1 Endocrinology, Diabetes and Metabolism, Shaukat Khanum Memorial Cancer Hospital and Research Centre, Lahore, PAK; 2 Diabetes & Endocrinology and Internal Medicine, Jersey General Hospital, Jersey, JEY; 3 Endocrinology and Diabetes, Shaukat Khanum Memorial Cancer Hospital and Research Centre, Lahore, PAK; 4 Endocrinology and Diabetes Mellitus , General (Internal) Medicine, Shaukat Khanum Memorial Cancer Hospital and Research Centre, Lahore, PAK; 5 Diabetes, Endocrinology and Metabolism, Shaukat Khanum Memorial Cancer Hospital and Research Centre, Lahore, PAK

**Keywords:** disorder of sex development (dsd), de la chapelle syndrome, testicular 46 xx karyotype, sry negative, hyper gonadotropic hypogonadism, azoospermia

## Abstract

Disorder of sex development (DSD) is the term ascribed to a wide group of disorders presenting with congenital discord between chromosomal sex and phenotypic manifestation. Its incidence is 1 in 4500 births. 46 XX testicular DSD is a rare disorder characterized by the discordance between female karyotype and male phenotype. Its incidence is 1:20,000 to 25,000 male infants. It is further classified into SRY positive and SRY negative individuals, depending on the presence or absence of sex-determining region Y gene (SRY) on the X chromosome as a result of translocation. We are hereby reporting a rare case of de la Chapelle syndrome (SRY negative).

A 30-year-old phenotypical male presented to us with complaints of primary infertility. He had had hypospadias during his childhood and underwent corrective surgery at the age of 18 years. For the previous 1.5 years, he had been complaining of decreased libido, difficulty in micturition, and presence of watery ejaculate. On examination, he had bilateral palpable testis with the testicular volume of 7 mL each, curved micropenis with chordee, and eccentric meatus with fistula. Semen analysis revealed azoospermia and hormonal profile was consistent with hypergonadotropic hypogonadism. His karyotyping turned out to be 46 XX chromosome without the SRY gene on polymerase chain reaction (PCR) array. He was medically treated with testosterone and underwent surgical correction of chordee.

The SRY negative testicular 46 XX disorder is a rare expression and can be diagnosed at the time of birth with the presence of severe hypospadias, cryptorchidism, or ambiguous genitalia. All new-borns with these findings should undergo evaluation for the disorder of sexual development. Such individuals can never father a child and genetic counseling should be offered. Infertility is the main concern for such individuals which can be addressed by in vitro fertilization (IVF) with a sperm donor or adoption.

## Introduction

Disorder of sex development (DSD) is the term first introduced in 2006 for infants born with ambiguous genitalia, or who have an appearance discordant with the chromosomal sex. Its incidence is 1 in 4500 births [1] and is usually caused by congenital adrenal hyperplasia, sex chromosome DSD (X/XY mosaicism), androgen insensitivity syndrome in XY individuals, XX testicular/Ovo testicular DSD, XY gonadal dysgenesis and many more [2]. 

The 46 XX testicular DSD is a rare case of gender dysplasia, also known as XX Male syndrome or de la Chapelle syndrome, named after de la Chapelle who first described it in 1964. Its incidence rate is 1 in 20,000 to 25,000. This karyotype may present clinically with normal genitalia, ambiguous genitalia or with the presence of both testicular and ovarian tissues simultaneously [3]. 

The Y chromosome in males usually contains a gene known as the sex-determining region Y gene (SRY) [4]. In 90% of 46 XX males, locus of SRY gene is shifted to the short arm of X chromosome or even to autosomes whereas 10% of such cases are just devoid of this gene altogether [5]. Some 85% of these individuals present at the time of puberty due to gynecomastia or for workup of infertility. Despite male phenotype, these males are sterile. They may have gynecomastia or small testicular size. Sterility is caused by azoospermia. Ambiguous genitalia are present in almost 15% of such cases at the time of birth. Their gender identity is male but in natural course experiences the consequences of testosterone deficiency. 

Here we report a rare case of such 46 XX male who did not have SRY gene expression. 

## Case presentation

A 30-year-old phenotypical male presented at an Endocrine Clinic for evaluation of primary infertility. His history dated back to the time of his birth when he had had hypospadias, for which he underwent surgery in Nigeria almost 12 years ago, at 18 years of age. Now for the last 1.5 years, he complains of low libido, difficulty in maintaining an erection with watery ejaculate, and difficulty in passing urine as well. 

On examination, his testes were palpable bilaterally with the volume of 7 mL. He had curved micropenis with chordee and eccentric meatus with fistula. He completed his investigations including a hormonal profile and semen analysis. Semen analysis revealed azoospermia and biochemical profile was consistent with hypergonadotropic hypogonadism. Table 1 illustrates his hormonal profile.

**Table 1:** Hormonal profile. FSH, follicle stimulating hormone; LH, luteinizing hormone

	Results	Normal range
Testosterone	7 nmol/L	7.6-31.4
FSH	26 IU/L	1.5-12.4
LH	16 IU/L	1.7-8.6

**Figure 1:**
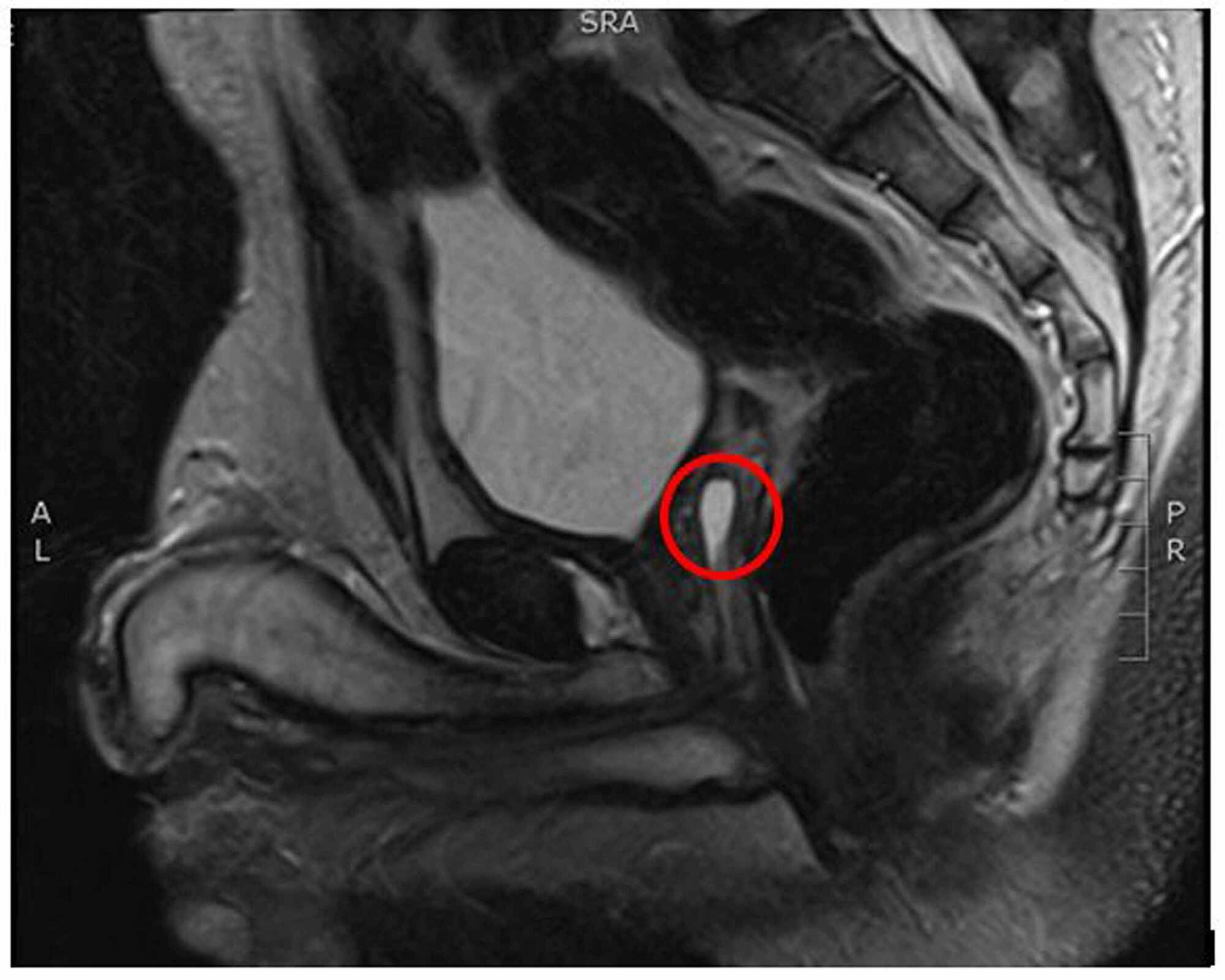
MRI abdomen and pelvis (red circle shows Mullerian duct remnant).

MRI abdomen and pelvis were performed which revealed Mullerian duct remnants (Figure 1). Testis were visible bilaterally in scrotum but were smaller in size.

In light of his clinical examination, phenotype and endocrine profile, chromosomal sex was determined via karyotyping. His chromosomal sex turned out to be 46 XX without mosaicism and we were unable to detect sex-determining region Y (SRY) gene via microdeletion polymerase chain reaction (PCR) array. As testosterone deficiency increases the risk of fragility fracture, so bone densitometry was performed that confirmed mild osteopenia. 

His discordance between phenotype and karyotype and the absence of SRY gene confirmed his diagnosis of 46 XX testicular syndrome. Ovo testicular variant of 46 XX DSD was excluded with the absence of ovaries on MRI scan. He was treated medically with testosterone replacement to avoid long-term complications of deficiency. He underwent another surgery for chordee and fistula. His case was under consideration for micro TESE although, much literature is not available in favor of this approach. 

## Discussion

Individuals with de la Chapelle syndrome may or may not have an SRY gene. In SRY positive individuals, testis determining factor (TDF) is produced, which is a gene regulatory protein whose expression results in inhibition of female sexual differentiation. Usually, this disorder is sporadic but few familial cases have also been documented. Amongst SRY positive group, most of the patients present in adolescence or adulthood with shorter than average height, gynecomastia, small testis, and azoospermia. Azoospermia is attributed due to lack of azoospermia factor region (AZF), whose locus is on the long arm of Y chromosome. 

It is hypothesized that in SRY negative group, implications of other genes linked to X chromosome are responsible for male phenotype such as SOX9, SOX3, SOX10, and RSOP1. A heterozygous gain of function mutations in NR5A1 causes inappropriate activation of testicular pathways. SOX 9 gene encodes a transcription factor that functions downstream of SRY and is also essential for testicular differentiation. SOX 3 does not appear to have any role in normal gonad development but once overexpressed, it activates testicular pathways. A few genes such as WNT4 and RSPO1’‘s mutations can also lead to testicular XX DSD. 

These individuals are born with ambiguous genitalia. They may have associated hypospadias with varying degrees of severity, chordee, and cryptorchidism [6]. Few cases with normal male external genitalia have also been reported. Hormonal evaluation reveals hypergonadotropic hypogonadism due to testicular failure. Human chorionic gonadotropin (hCG) stimulation test can be performed which exhibits the failure of testosterone to rise after administration of hCG. Radiological studies are performed to find the presence of Mullerian ducts remnants if any. Their removal may be necessitated because of them posing a risk of future infections [7]. The karyotype is determined and molecular genetic testing for detection of SRY gene is done via fluorescence in situ hybridization (FISH). Failure to detect SRY by FISH may benefit from chromosomal microarray (CMA) which can detect SRY including mosaicism.

**Figure 2:**
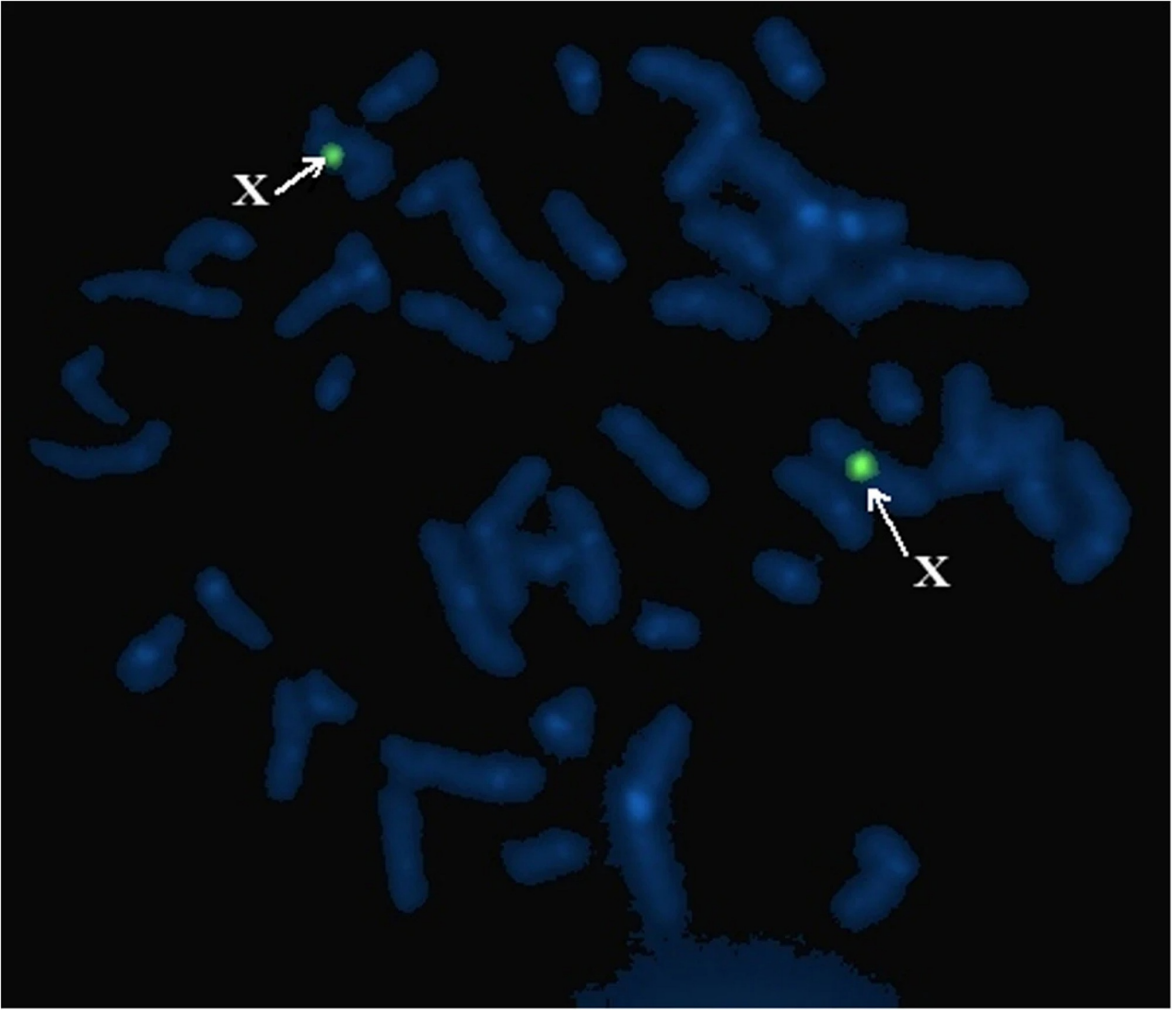
FISH: SRY negative individuals. FISH, fluorescent in situ hybridization

**Figure 3:**
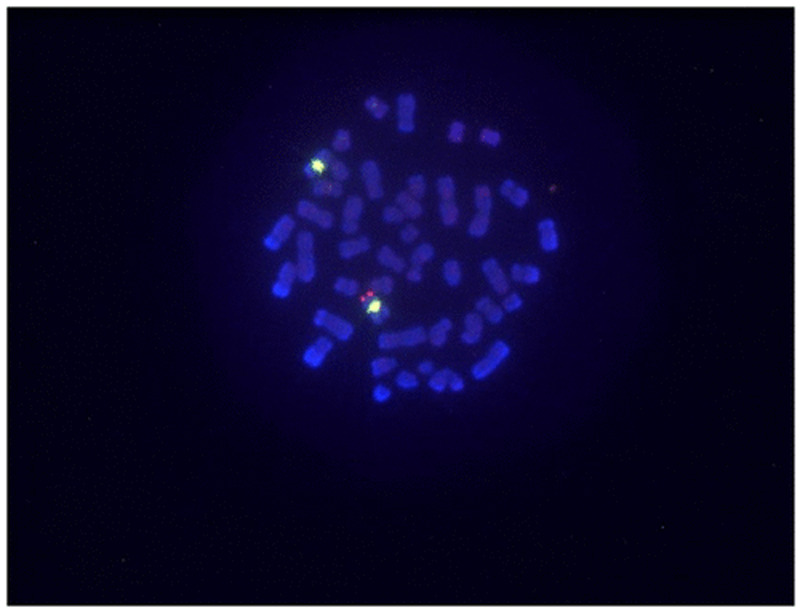
FISH: SRY positive individuals. FISH, fluorescent in situ hybridization

Figures 2 and 3 show FISH analysis in two different cases with SRY negative [8] and positive [9] karyotype. 

The natural course of this disorder includes typical outcomes from testosterone deficiency; lack of libido, erectile dysfunction, decrease in secondary sexual characteristics, osteopenia, compromised muscular strength, and depression [10]. Bone densitometry is done in these individuals to assess for risk of fragility fractures. Testosterone is replaced to avoid possible complications. Unfortunately, these individuals cannot father a child so they should be offered in vitro fertilization (IVF) with a sperm donor or adoption [11] if they seek advice regarding infertility.

Research is still ongoing on modes of inheritance of SRY negative variant of 46, XX testicular DSD. Some suggest it is autosomal recessive. Inheritance related to SOX9 gene is autosomal dominant but that of SOX3 is still unknown. 

## Conclusions

The presence of certain specific clinical features even at the time of birth or during early years of life is suggestive of this rare diagnosis. The early diagnosis would still not allow cure of this condition with the currently available treatment options. However, it can potentially have significant implications on personal, social, and married lives of these individuals. Clinicians should keep a high index of suspicion of this and other DSD for early diagnosis especially in the presence of specific relevant clinical features. 
